# Changes in arterial cerebral blood volume during lower body negative pressure measured with MRI

**DOI:** 10.1016/j.neuroimage.2017.06.041

**Published:** 2019-02-15

**Authors:** Joseph R. Whittaker, Molly G. Bright, Ian D. Driver, Adele Babic, Sharmila Khot, Kevin Murphy

**Affiliations:** a Cardiff University Brain Research Imaging Centre (CUBRIC), School of Psychology, Cardiff University, Cardiff CF24 4HQ, United Kingdom; b Cardiff University Brain Research Imaging Centre (CUBRIC), School of Physics and Astronomy, Cardiff University, Queen's Buildings, The Parade, Cardiff CF24 3AA, United Kingdom; c Sir Peter Mansfield Imaging Centre, School of Medicine, University of Nottingham, Nottingham NG7 2RD, United Kingdom; d Division of Clinical Neurosciences, School of Medicine, University of Nottingham, Nottingham NG7 2UH, United Kingdom; e Department of Anaesthesia and Intensive Care Medicine, Cardiff University School of Medicine, Cardiff CF14 4XN, United Kingdom

**Keywords:** Arterial spin labeling, Cerebral autoregulation, Cerebral blood volume, Lower body negative pressure, Magnetic resonance imaging

## Abstract

Cerebral Autoregulation (CA), defined as the ability of the cerebral vasculature to maintain stable levels of blood flow despite changes in systemic blood pressure, is a critical factor in neurophysiological health. Magnetic resonance imaging (MRI) is a powerful technique for investigating cerebrovascular function, offering high spatial resolution and wide fields of view (FOV), yet it is relatively underutilized as a tool for assessment of CA. The aim of this study was to demonstrate the potential of using MRI to measure changes in cerebrovascular resistance in response to lower body negative pressure (LBNP). A Pulsed Arterial Spin Labeling (PASL) approach with short inversion times (TI) was used to estimate cerebral arterial blood volume (CBV_a_) in eight healthy subjects at baseline and −40 mmHg LBNP. We estimated group mean CBV_a_ values of 3.13 ± 1.00 and 2.70 ± 0.38 for *baseline* and *lbnp* respectively, which were the result of a differential change in CBV_a_ during −40 mmHg LBNP that was dependent on baseline CBV_a_. These data suggest that the PASL CBV_a_ estimates are sensitive to the complex cerebrovascular response that occurs during the moderate orthostatic challenge delivered by LBNP, which we speculatively propose may involve differential changes in vascular tone within different segments of the arterial vasculature. These novel data provide invaluable insight into the mechanisms that regulate perfusion of the brain, and establishes the use of MRI as a tool for studying CA in more detail.

## Introduction

It is vital for neurological health that cerebral blood flow (CBF) is kept above a minimum threshold to sustain the high metabolic rate of the human brain (Van Lieshout et al., 2003). CBF, which is defined as the rate of blood delivery to brain tissue, is primarily determined by the ratio of cerebral perfusion pressure (CPP) and the combined vascular resistance between the heart and the capillary bed. Resistance to flow is inversely proportional to vessel lumen diameter, and so can be dynamically modulated via the action of smooth muscle lined cerebral arterioles in order to support metabolic activity locally. Furthermore, the degree to which upstream arterioles dilate in response to a vasoactive stimulus is a marker of local cerebral haemodynamic functional integrity, termed cerebrovascular reactivity (CVR). The systemic protective process that keep CBF stable in the presence of fluctuations in the cardiovascular system (cardiac output and systemic blood pressure) is known as cerebral autoregulation (CA). Although regulation of blood flow is affected by numerous physiological mechanisms, this definition is usually restricted to those concerning systemic blood properties. Thus, both CVR and CA form a complementary basis on which to understand systemic regulation of blood flow in the brain, and impairment within either of these domains will have clinical implications.

Transcranial Doppler (TCD) ultrasonography is often relied upon as a cost effective means of assessing CVR and CA in real time with a high temporal resolution, in both research and clinical practice (Purkayastha and Sorond, 2012, Willie et al., 2011). However, due to associated high error rates and its operator dependent nature, more recently functional Magnetic Resonance Imaging (fMRI) has emerged as a tool for measuring CVR (Heyn et al., 2010, Pillai and Mikulis, 2015, Shiino et al., 2003). The advantage of MRI over TCD is that it allows CVR to be mapped across the whole brain with high spatial resolution. Although CA challenges exist and are widely employed in TCD-based physiology research, the use of MRI as a tool to measure the cerebrovascular responses associated with CA has been limited. Nevertheless, it would be desirable to develop MRI based approaches for studying CA function for the same reasons that apply to CVR research, namely the excellent spatial resolution and whole-brain field-of-view (FOV) afforded by MRI, but also sensitivity to multiple different flow-dependent image contrast mechanisms that allow a variety of research questions to be addressed.

For clinical assessment of CA gravitational stressors such as tilt-table tests (Heckmann et al., 1999) and orthostasis (Levine et al., 1994) (standing) are most often used, because they are relatively easy to implement in the laboratory and have real world relevance. An alternative technique that can be used to simulate gravitational shifts in central blood volume, such as those that accompany orthostasis, is Lower Body Negative Pressure (LBNP) (Lundvall and Edfeldt, 1992). As the subject remains in the supine position during the LBNP challenge this makes it suitable for use in the MR environment, however to date, there have been relatively few attempts to combine MRI and LBNP to study CA. To administer LBNP, participants lie in the supine position with their legs placed in a chamber that is sealed at the level of the iliac crest, and the air pressure inside is subsequently lowered using a vacuum source. This makes the pressure inside the chamber lower than the atmospheric pressure experienced by the upper body, and thus in accordance with the laws of fluid dynamics, blood volume is redistributed to the area of lower pressure (i.e. the legs in the chamber). LBNP is typically administered in a step-wise approach of graded decreases in pressure, and it is generally accepted that this results in progressive reductions in central venous pressure, cardiac output and increases in total peripheral resistance and heart rate respectively (Wolthuis et al., 1974). How mean arterial pressure (MAP) responds to progressive LBNP is less clear, but most studies report no change for milder levels of LBNP (up to −20 mmHg) (Brown et al., 2003, Hisdal et al., 2001, Lundvall and Edfeldt, 1992, Serrador et al., 2000), with some reports of small decreases in response to larger negative pressure changes (Halliwill et al., 1998, Pannier et al., 1995, Rickards et al., 2015), but not exclusively (Brown et al., 2003, Serrador et al., 2000).

The aim of this study was to evaluate the combined use of MRI and LBNP as a tool for probing the mechanisms that underlie CA, using an MRI-compatible LBNP chamber to deliver an orthostatic challenge to a group of healthy participants in the MRI scanner. Changes to cerebrovascular resistance occur via changes in vessel diameter, but measuring this process directly with MRI is currently only possible in the larger arteries of the brain, and is usually performed at high field strengths (> 3 T) (Park et al., 2017). However, given that these changes necessarily occur on the arterial side of vasculature, arterial cerebral blood volume (CBV_a_) can provide a surrogate measure that is indirectly sensitive to these changes. In this study, we used an Arterial Spin Labeling (ASL) based approach to estimate CBV_a_, and found a baseline dependent response during LBNP.

## Methods and materials

### Theory

Measuring CBV with MRI without the use of contrast agents is not trivial and there is no gold-standard approach. BOLD fMRI methods exist, but are intrinsically more weighted towards the venous (deoxygenated) blood volume compartment (Blockley et al., 2013). ASL is a well-established MRI method that utilises arterial blood water as an endogenous tracer for non-invasive quantification of CBF, but which can also be used to obtain a measure of arterial CBV (CBV_a_) by waiting only a short time after tagging to acquire an image (Brookes et al., 2007, Chappell et al., 2010, Francis et al., 2008, Petersen et al., 2006, Warnert et al., 2015b). When a short inversion time (TI) is used, tagged blood water spins do not have sufficient time to flow to the capillary bed, and are thus still confined to the arterial vasculature (Chappell et al., 2010). Using a very short echo time (TE) to minimise the effect of an unknown arterial blood transverse relaxation rate, the measured difference signal is then simply the remaining longitudinal magnetisation of the arterial blood volume fraction of a voxel.

The general kinetic model of the ASL difference signal (ΔM) proposed by Buxton et al., is derived from an arterial input function, a residue function, and a magnetization relaxation function (Buxton et al., 1998). These functions respectively model the arrival of the tagged water spins to the imaging voxel, the fraction of tagged water spins that remain at time *t* after arrival, and the decrease in signal at time *t* due to longitudinal relaxation of tagged water spins. When a sufficiently short TI is used, such that the ΔM signal is completely intravascular, it can be modelled using the arterial input function (Chappell et al., 2010, Van Osch et al., 2006), which for PASL data is given by:

(1) $$\Delta M=\{\begin{array}{cc} 0 & t<\mathrm{BAT}\\ 2\alpha M_{0a}e^{-t/T_{1a}}{\mathrm{CBV}}_{a} & \mathrm{BAT}\leq t\leq \mathrm{BAT}+\tau \\ 0 & \mathrm{BAT}+\tau \leq t \end{array}$$

where M_0a_ and T_1a_ are the equilibrium magnetisation and longitudinal relaxation time of arterial blood respectively, α is the tagging efficiency, BAT is the bolus arrival time, τ is the temporal width of the tag, and CBV_a_ is the fraction of voxel volume occupied by arterial blood.

Eq. (1) shows that the ASL difference signal is sensitive to CBV_a_ when BAT<TI≤BAT+τ. This is assuming that no exchange of tagged water spins with untagged tissue spins occurs (i.e. artery walls are impermeable), and that blood passes instantaneously through the voxel (Chappell et al., 2010). These assumptions are only valid for larger arterioles with sufficiently high flow velocities, and so this approach is only suitable for estimating blood volume in large arteries/arterioles. In practice, choosing a TI that satisfies the above condition is not straight forward, and the choice of TI will determine the sensitivity of the signal to arteries of different sizes. It is therefore desirable to measure the signal from multiple short TIs and to fit the measured signal curve to the model to obtain a measure of CBV_a_ that is insensitive to the arrival time of tagged blood.

Another factor that confounds the estimation of CBV_a_ is dispersion of the tagged bolus between the time of inversion and the time it reaches the imaging slice. The model in Eq. (1) assumes a simplistic “plug” flow profile, i.e. a uniform cross-section of flow velocities. It can be extended to included dispersion of the tag arrival times as would be expected of a more realistic laminar flow-profile (Wu et al., 2007). Assuming a Gaussian distribution of bolus arrival times (δt~N(BAT,σ)), with a standard deviation σ representing the degree of temporal dispersion, the arterial input function is now modelled as:

(2) $$\Delta M=2\alpha M_{0a}{\mathrm{CBV}}_{a}e^{-t/T_{1a}}\left[w(t)⋇\delta t\right]$$

where w(t) is a square function given by:

(3) $$w\left(t\right)=\{\begin{array}{cc} 1 & 0\leq t\leq \tau \\ 0 & \tau <t \end{array}$$

Throughout, we refer to the simpler model of Eq. (1) as the *plug-flow* model, and the more complex model of Eq. (2) as the *laminar-flow* model. Fig. 2A shows simulated ∆M curves for both the *plug-flow* and *laminar-flow* models.

### Experimental protocol

#### Subjects

Eight healthy adult male subjects participated in the study. All participants gave written informed consent, and the School of Psychology Cardiff University Ethics Committee approved the study in accordance with the guidelines stated in the Cardiff University Research Framework (version 4.0, 2010). To simplify the study only male subjects were included, as differences in orthostatic tolerance between sexes (Russomano et al., 2015) would increase inter-subject variability in a mixed subject sample.

#### Magnetic resonance imaging acquisition

A 3T GE HDx scanner equipped with a body transmit and eight-channel receiver head coil was used to acquire images using a PICORE Pulsed Arterial Spin Labeling (PASL) sequence (Wong et al., 1997), with a gradient-echo spiral readout at four short inversion times (TI_1_/TI_2_/TI_3_/TI_4_ = 150,300,450,600 ms, TE = 3 ms, TR=variable, FOV = 220 mm, 64 × 64 matrix (~ 3.4 mm^2^ in-plane resolution), 12 slices (7 mm thick + 1 mm gap), 20 cm tag width, 1 cm tag/slice gap, 40 tag/control pairs per TI). To estimate the equilibrium magnetization (M_0_) of arterial blood, a single echo scan was acquired with the same parameters as above, but minus the ASL tag preparation. A minimum contrast scan (TE/TR = 11/2000 ms) was also acquired to correct for field inhomogeneity.

During all runs pulse waveforms and oxygen saturation (SO_2_) were recorded (Medrad, PA, USA), blood pressure measurements were collected using an arm-cuff at 1-min intervals (OMRON, Tokyo, Japan). Expired gas content was recorded (AEI Technologies, PA, USA) and sampled at 500 Hz (CED, Cambridge, UK) to obtain measures of partial pressure of end-tidal respiratory carbon dioxide (P_ET_CO_2_).

#### Lower body negative pressure chamber

A wooden (MRI compatible) semi-cylindrical LBNP chamber fitted with a variable vacuum source was built in-house (see Supplementary material Fig. 1). The chamber consisted of a separate base and (semi-cylindrical) cover, allowing it to be assembled in the MRI environment. The chamber base was placed on the scanner bed so that when subjects then lay in the supine position, the cover could fit to the base to form a loosely sealed container of the lower body. The chamber was then completely air tight sealed at the iliac crest with disposable plastic wrap, allowing stable LBNP levels to be maintained. A manometer was used to gauge the chamber pressure, which was lowered using the variable vacuum source.

The PASL sequence was used to acquire data for each subject whilst placed inside the LBNP chamber. Two runs of data were collected for each subject; a run without any applied vacuum, i.e. at atmospheric pressure (*baseline run*), and a run with the vacuum applied to achieve a pressure of 40 mmHg below atmospheric (*lbnp run*). All subjects completed a “mock” session under the supervision of a clinician (consultant anesthetist) prior to the scanning session, to prove they could tolerate LBNP at the required level without any indication of pre-syncope. This involved lowering the pressure to 40 mmHg below atmospheric level in increments of 10 mmHg. For each increment, the pressure was lowered over the course of a minute, and then kept at a stable level for a further minute. All subjects tolerated this and no symptoms of pre-syncope were reported.

For the scanning session, subjects were sealed in the LBNP chamber, which was then slowly brought down to −40 mmHg to test the seal. For *lbnp* runs, the pressure was brought slowly down to the required level over the course of a minute. Although within scan subject motion was not significantly modulated by LBNP, the relative vacuum created at −40 mmHg caused subjects to move into the chamber (~ 2 cm). For this reason, imaging volumes were positioned separately for each condition. As imaging volumes were positioned separately for each condition, shimming was also performed separately. A clinician was present for all scan sessions to ensure participant safety and to terminate the scan if any predefined stopping criteria indicative of pre-syncope were met. All subjects completed all scans and reported no negative symptoms.

### Data processing

#### Preprocessing

Data were analysed in AFNI (Cox, 1996) and FSL (Jenkinson et al., 2012). To correct for motion within each condition, images were registered to the mean volume of the condition, and then co-registered to the same space. Framewise displacement (FD) was estimated using the sum of the absolute values of the first derivative of realignment parameters, and the mean was used as a summary statistic to characterise the degree of motion related variance expected for each run. To avoid any bias that may arise from asymmetry in the co-registration (i.e. by keeping one image fixed as a reference), we used the matrix square root of an affine transformation to define a “mid-point” between the two conditions. This procedure is recommended for applications such as this (Smith et al., 2001), as it ensures the degree of interpolation-related blurring is approximately the same for both conditions. For each run, voxel-wise time-series were created with four data points, corresponding to the average tag/control difference (ΔM) for each TI. For the subsequent analysis, a mask was created to identify voxels with a significantly large arterial weighting, i.e. large difference signal across TIs (see below for details). As both runs were registered to the same space, a single mask for each subject was used, which allowed the same regions of the brain to be compared during *baseline* and *lbnp* runs, within the limits of the relatively course spatial resolution.

#### Arterial mask definition

**Fig. 1:**
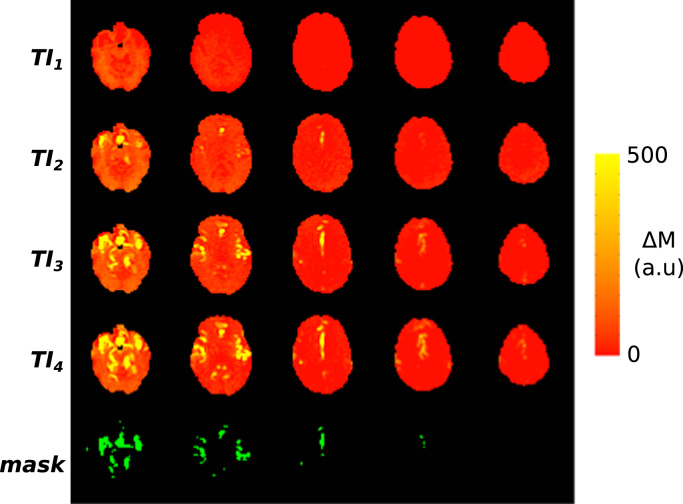
∆M maps for a representative subject (1) and corresponding macro-vascular mask. The lack of contrast in much of the image reflects the sparse nature of the large arterial vasculature that the method is sensitive to.

A macro-vascular mask was created for each subject and each condition in order to identify voxels in which the arterial vasculature has been filled with tagged blood, i.e. with a large ΔM signal. As this depends on arrival time, data from all TIs was combined in such a way to remove this confound. The mask was generated for each subject and condition as follows; for each run of data, each TI image was normalised to correct for T_1_ relaxation using an assumed T_1_ of blood (1.7 s), also accounting for slice timing. Thus, new corrected time series data were created, in which theoretically, only tagged blood volume signal variations should be present. The mean image across TIs in this new dataset was calculated (excluding the first TI as it is expected to be too soon for the bulk of the tagged bolus to have arrived in the imaging volume), and voxels above the 95 percentile were selected to be in the mask. Using these criteria, the mask should contain only those voxels with a significantly large arterial contribution, and should be relatively insensitive to arrival time. As this mask specification procedure was performed on data for both conditions, two masks per subject were created (*baseline* and *lbnp*). The intersection of the *baseline* and *lbnp* masks was then calculated in order to create a single mask without bias towards either condition. All subsequent processing of ASL data was restricted to voxels within the mask. Fig. 1 shows an example of the data across TIs and of the macro-vascular mask.

#### CBV_a_ estimation

**Fig. 2:**
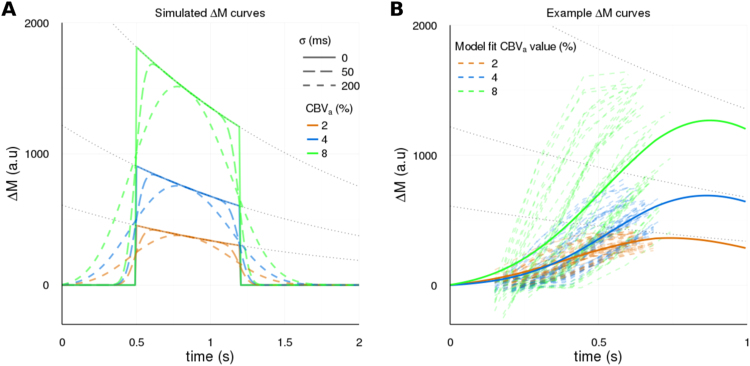
A) Simulated ∆M curves for a mean BAT of 0.5 s. B) Example ∆M curves (top 50 R^2^ values for each CBV_a_ value) from a representative subject (1), colour coded according to their assigned CBV_a_ value in the *laminar-flow* model fit (for illustration purposes only time course that were assigned CBV_a_ parameter estimates of 2,4,or 8% are shown). Solid lines show the mean fit across voxels, which are smoothed out due to the presence of multiple different BAT and σ values).

The *laminar-flow* (with dispersion parameter σ) and *plug-flow* (without dispersion parameter σ) intravascular signal models were fit to all voxels within the mask, using a template-based brute-force approach in the *nls2* package (Grothendieck, 2013) in R Core Team (2015). This approach has previously been validated for PASL signal time curve data (Wu et al., 2007), and was chosen to overcome the challenge of fitting non-linear models to a small number of data points. A template of different ΔM signal time curves was generated using an array of fixed discrete parameter values (with a physiologically realistic range); CBV_a_(%) = [0,1, 2, …, 10], BAT(s) = [0.1,0.2,0.3, …, 1] and σ(s) = [0,0.2,0.4]s (i.e. “plug” flow, or 200/400 ms temporal dispersion of δt in Eq. (2)). Note that 0% was the minimum CBV_a_ parameter value considered, allowing for the possibility of voxels with no delivery of tagged blood being present, which in practice is unlikely due to the mask inclusion criteria (see above). Fig. 2B shows example ∆M curves from a representative subject, that are colour coded according to the CBV_a_ parameter value that best fits them in the laminar flow model.

All combinations of these template signal time curves were estimated for both flow profiles (i.e. σ=0 and σ=0.2/0.4 s), and those that minimised the residual sum of squares (RSS) were selected as the best-fit models. The best-fit model for each flow-profile was determined using the Akaike Information Criterion (AIC). Thus, every voxel in the mask was characterised by a set of parameter values (CBV_a_, BAT, and σ), and a R^2^ value to determine the quality of the model fit. Additionally, the *plug-flow* model estimates were also considered separately, so that every voxel was characterised by only CBV_a_, and BAT and R^2^. For the longitudinal relaxation time of arterial blood, a literature value (Hales et al., 2016) was used (T_1a_ = 1.7 s). The equilibrium magnetisation of arterial blood (M_0a_) was estimated using a measurement of CSF M_0_ (M_0CSF_) derived from the lateral ventricles of the single echo M_0_ scan (Liau et al., 2008), and the tagging efficiency was assumed to be α = 1.

This brute-force template based model fitting approach has previously been validated for ASL data acquired with the same PICORE sequence, albeit with more conventional longer TI times (Wu et al., 2007). We repeated the same numerical simulations in order to examine the validity of this approach for short TI times, show in Appendix A. The simulations reveal an overall error rate < 1% CBV_a,_ which means the model fit approach can robustly estimate this parameter within the discrete resolution we have defined. Although these numerical simulations suggest we are able to robustly estimate CBVa using the model based approach, we also considered a naïve non model derived estimates of CBV_a_ to rule out any effect of model misspecification. As stated in the theory section, the ∆M signal is sensitive to CBV_a_ when BAT<TI≤BAT+τ. We therefore used the ∆M signal for TI_4_ to estimate CBV_a_ on a continuous scale according to Eq. (1) (*TI*_*4*_ *estimate*), assuming that 600 ms is long enough to allow the bulk of the tagged bolus to have arrived in all the arteries we are sensitive to.

### Statistical analysis

Statistical analysis was performed on the model estimate parameter values. For each subject the mean parameter estimate across voxels was calculated for both conditions, and then paired *t*-tests were used to look for significant change during LBNP. To explore the data in more detail the difference in parameter values (∆BAT, ∆σ) between runs (*baseline* - *lbnp*) was calculated on a voxel-wise basis, and for CBV_a_ the difference between conditions was expressed as a fraction of the *baseline* value (%∆CBV_a_ = 100*(CBV_a_^lbnp^ - CBV_a_^*baseline*^)/CBV_a_^*baseline*^). To look for more complex trends in the data, the differences in parameter values between conditions (%∆CBV_a_, ∆BAT, ∆σ) were then grouped according to CBV_a_^baseline^ and then combined across subjects into a single vector of numbers (N = 80, n = 8 subjects and 10 CBV_a_ baseline values).

For each parameter of interest, a linear mixed effects (LME) model was fit to this vector of parameter difference estimates using the *lme4* package (Douglas Bates et al., 2015) in R, including random effects terms to control for within subject correlations, and a fixed effect of *baseline* CBV_a_ (i.e., a random intercept model). By comparing with a null model of subject specific means, this allows us to test for an effect of *baseline* CBV_a_ dependency on parameter changes between conditions.

## Results

**Fig. 3:**
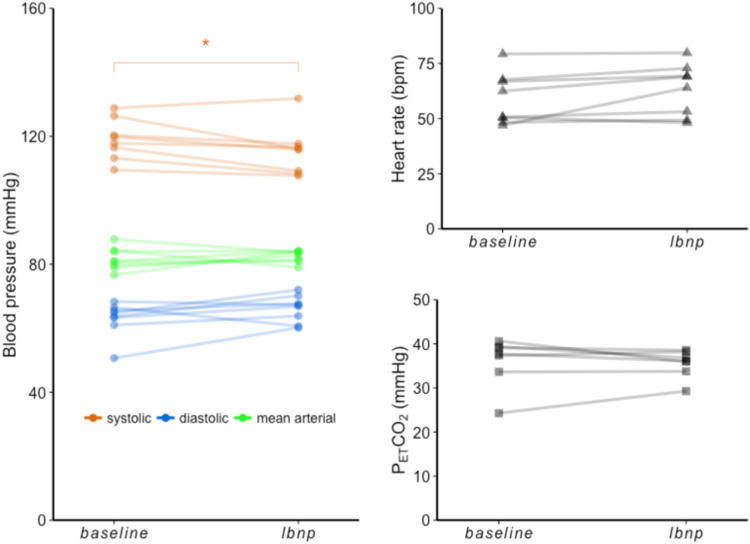
Plots to show systolic, diastolic and mean arterial blood pressure, heart rate, and P_ET_CO_2_ at *baseline* and *lbnp*. * denotes significant changes (p<0.05).

The physiological responses to the LBNP challenge are presented in Table 1 and Fig. 3. The most apparent systemic changes were to systolic blood pressure and HR, which saw a modest decrease in all but one subject (mean (±SD) = −3.64 ± 4.04 mmHg, p = 0.038) and a trend towards an increase (mean (±SD) = 4.10 ± 5.88 bpm, p = 0.089) respectively in most subjects during *lbnp*. No significant change in MAP was found, indicating the presence of a compensatory increase in peripheral resistance. There was no significant change in average P_ET_CO_2_ recorded for each condition, which is important given that CO_2_ is a strong vasodilator that previously has been suggested to be a confounding factor in blood flow measurements made during LBNP (Brown et al., 2003). Table 1 also lists values of M_0a_ estimated for each condition, and M_0a_-normalised mask-averaged ΔM values (ΔM/M_0a_) for each TI. There was no systematic variation in estimated M_0a_ values between conditions that could confound subsequent CBV_a_ estimates. Mean FD was 0.056 mm and 0.077 mm for *baseline* and *lbnp* runs respectively (non-significant, p=0.204), suggesting motion also did not present a major confound in the subsequent analysis. A pairwise *t*-test revealed a significant difference between conditions in the mask-averaged ΔM/M_0a_ for the 150 ms TI.

**Table 1:** Responses to LBNP. Values are mean ± SD. Group mean changes in recorded physiological parameters, normalised difference signals per TI, and model parameter estimates are show.

Condition	Blood pressure (mmHg)					Heart rate (bpm)		P_ET_CO_2_ (mmHg)
	Systolic	Diastolic		MAP				
*baseline*	119 ± 6.38*	63.0 ± 5.44		81.7 ± 3.53		59.1 ± 11.7		36.0 ± 5.63
*lbnp*	115 ± 7.80*	66.1 ± 4.24		82.5 ± 1.87		63.2 ± 11.7		35.5 ± 3.17

	
	ΔM/M_0a_							
	TI1 (0.15 s)		TI2 (0.3 s)		TI3 (0.45 s)		TI4 (0.6 s)	

*baseline*	−0.35 ± 0.28†		1.31 ± 1.10		3.19 ± 1.47		4.36 ± 1.35	
*lbnp*	−0.07 ± 0.35†		1.22 ± 0.83		2.87 ± 0.79		3.81 ± 0.61	

	
	Model estimates							
	CBVa (%)		BAT (s)		σ (s)		M_0a_ (a.u)	

*baseline*	3.13 ± 1.00		0.58 ± 0.08		0.15 ± 0.03		1.70 ± 0.15	
*lbnp*	2.70 ± 0.38		0.57 ± 0.05		0.18 ± 0.03		1.73 ± 0.23	

* p < 0.05 (uncorrected) compared with *baseline*.

† p < 0.05 (Bonferroni corrected) compared with *baseline*.

### CBV_a_ estimates

Group mean mask averaged model estimate CBV_a_ values were 3.13 ± 1.00 and 2.70 ± 0.38 for *baseline* and *lbnp* respectively, which is in good agreement with ASL based literature values of pre-capillary arterial vasculature (Brookes et al., 2007). This small reduction in mean CBV_a_ during *lbnp* was not significant. Mean BAT was almost identical during *baseline* and *lbnp*, with estimates of 0.58 ± 0.08 and 0.57 ± 0.05 respectively, with no significant difference, and is consistent with expected times for tagged blood to arrive in the cerebral vasculature (Chappell et al., 2010). The mean tag dispersion σ was very similar for *baseline* and *lbnp*, 1.70 ± 0.15 and 1.73 ± 0.23 respectively, with no significant difference.

**Fig. 4:**
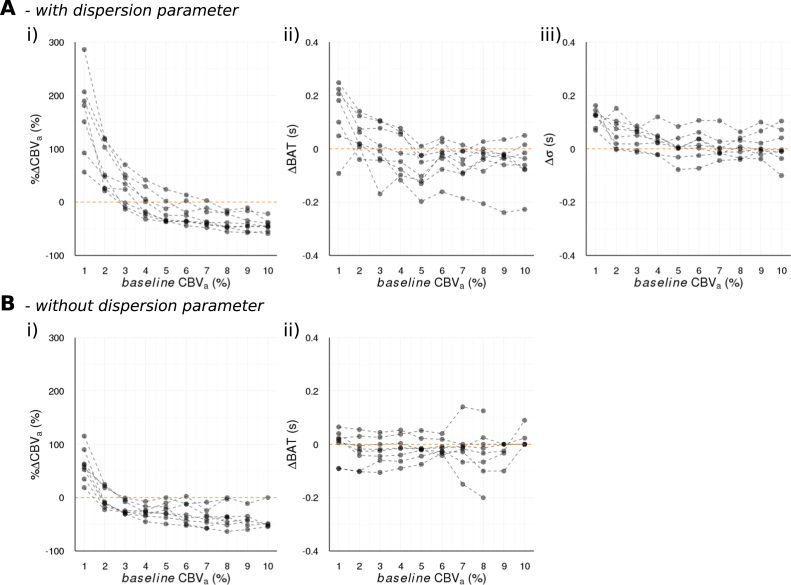
Individual subject changes in estimated parameter values as a function of the estimated *baseline* arterial cerebral blood volume (CBV_a_) for A) *laminar-flow* model and B) *plug-flow* model. Parameters are, i) fractional change in CBV_a_ during *lbnp* (%∆CBV_a_), ii) bolus arrival time (BAT) in absolute units of seconds, iii) temporal dispersion of tagged blood bolus (σ) in absolute units of seconds.

Fig. 4 shows model parameter changes, as a function of CBV_a_^baseline^, for the *laminar-flow* model with the dispersion parameter, and without in the simpler *plug-flow* model for comparison. Most strikingly, it can be seen that the %∆CBV_a_ strongly depends on the *baseline* value, and this observed inverse relationship between %∆CBV_a_ and CBV_a_^baseline^ is not contingent on assuming any particular flow profile, which suggests it is a robust result and not the result of overfitting.

The LME model including an effect of CBV_a_^baseline^ explained significantly more of the variance in %∆CBV_a_ (likelihood ratio = 39.3, p ~ 10^–12^), but not in ∆BAT (likelihood ratio = 1.54, p = 0.21), or ∆σ (likelihood ratio = 3.32, p = 0.07). This demonstrates a large effect of the CBV_a_^baseline^ value on %∆CBV_a_.

### Single TI CBV_a_ estimate

**Fig. 5:**
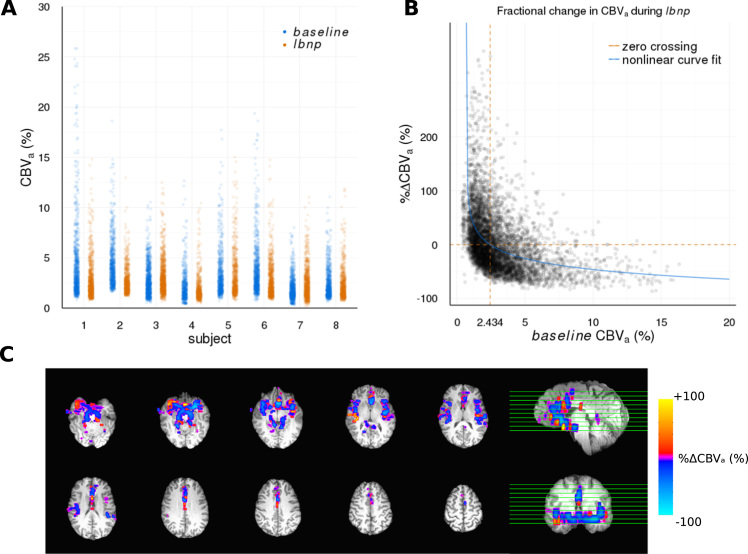
A) Continuous *TI*_*4*_*estimate* voxel-wise CBV_a_ values for *baseline* and *lbnp* conditions for each subject. B) Fractional change in continuous *TI*_*4*_*estimated* CBV_a_ during *lbnp* (%∆CBV_a_) as a function of *baseline* CBV_a_, pooled across all subjects. Nonlinear curve fit of the form a(x-b)^c^+d is plotted to show the inverse trend in the data. C) Representative subject (1) %∆CBV_a_ map.

Fig. 5A shows *TI*_*4*_ *estimates* of CBV_a_ for both *baseline* and *lbnp* conditions, and Fig. 5B %∆CBV_a_ plotted against CBV_a_^baseline^ pooled across all subjects (*individual subject plots are shown in* Supplementary material Fig. 2). The same inverse relationship is present, suggesting it is not the result of any bias in model fitting between the *baseline* and *lbnp* conditions. Fig. 5C shows a *TI*_*4*_ *estimate* %∆CBV_a_ parameter map for a representative subject (*individual subject plots are shown in* Supplementary material Fig. 3).

## Discussion

This study has demonstrated the feasibility of combining LBNP and MRI to non-invasively investigate CA. Our main finding is that LBNP caused changes to CBV_a_ that are differentially determined by the *baseline* value.

### CBV_a_ response to LBNP

TCD measured reductions in cerebral artery blood flow velocity (CBFV) within large arteries have been reported consistently (Bondar et al., 1994, Brown et al., 2003, Giller et al., 1992, Larsen et al., 1994). This apparent increase in cerebrovascular resistance (and thus reduction in CBF) is hard to reconcile with the traditional notion of CA, in which CBF would be expected to remain stable, and suggests some degree of vasoconstriction occurs in the brain as well as the periphery. The finding of a reduction in CBV_a_ in those voxels with larger *baseline* values is indicative of vasoconstriction occurring somewhere within the arterial vasculature, and so is consistent with this TCD literature.

The finding of a robust *baseline* dependent change in CBV_a_ during LBNP is an intriguing one, which suggests a complex and possibly divergent vascular response. As volume is proportional to vessel diameter, changes in CBV_a_ can be explained in terms of changes to vascular tone. Without an independent measure of vessel type, or an ability to effectively separate the contribution of different vessels to our CBV_a_ measurements we must be cautious in our interpretation of these data. However, given what is known about the cerebral vasculature, we believe the most plausible explanation for these data is a vascular response to the LBNP challenge that is subject to vessel size, which we discuss in detail in the following section.

#### Vessel size dependent response?

The architecture of the cerebral vasculature is organized such that the main arteries stemming from the CoW divide into progressively smaller pial arteries/arterioles that run across the cortical surface and eventually penetrate the brain parenchyma (Cipolla, 2009). These penetrating arterioles bifurcate into gradually narrower branches that ultimately supply the capillary bed of each cortical layer with a given area of cortex. This arrangement means that cerebral vessel diameters span several orders of magnitude, from the largest arteries to the capillaries, and so CBV estimates from a single voxel containing both large pial arteries and small capillaries are weighted towards the largest vessel. However, it should be noted that for these ASL data we are only sensitive to arterial vessels that have had time to fill with tagged blood within the inversion times used. As we have a relatively course spatial resolution, all voxels likely contain a mixture of vessel types, but only those with sufficiently early arrival times will contribute to the measure signal.

Thus, if a voxel containing multiple vessel sizes can be described by a skewed distribution of CBV values, the mean value is a reasonable approximation of vessel size, but also serves as an upper bound in the (unlikely) instance of a voxel containing a single vessel. This argument is supported by experiments utilizing iron oxide as an intravascular contrast agent to estimate both CBV and vessel size index (VSI) via changes in the apparent transverse relaxation time T_2_* (Kim et al., 2013). In these experiments both VSI and CBV show a similar dependence on cortical depth, implying one is a reasonable approximation of the other. If we consider CBV_a_ to be a surrogate measure for vessel size, then given that a fractional change in blood volume is proportional to the fractional change in radius squared, one possible explanation for the *baseline* dependent CBV_a_ effect is that there is a vessel size dependent effect of LBNP. This implies constriction of larger arterial vessels and dilation of smaller ones. Because constriction of large arteries will increase cerebrovascular resistance and reduce flow velocity, this interpretation is compatible with existing TCD literature, in which CBFV (typically in the MCA) is consistently reported to decrease during LBNP challenges.

Exactly why larger cerebral arterial vessels should constrict in response to the LBNP challenge is not clear. However, one possible explanation arises from the known increase in sympathetic nervous activity (SNA) that occurs during LBNP (Joyner et al., 1990). Perivascular nerve fibres are present in the walls of brain vessels, which would logically imply some vasoactive function, yet historically the role of neural control in CA has been the subject of intense debate, with inconsistent findings presented in existing animal studies (Ainslie and Brassard, 2014, Sandor, 1999, Strandgaard and Sigurdsson, 2008, Van Lieshout and Secher, 2008, Brassard et al., 2017). However, several human studies have found evidence to suggest SNA does influence vascular tone and thus may participate in CA, if only moderately (Jordan et al., 2000). Wilson et al. used both LBNP and a cold pressor test to increase SNA, but with differential effects on blood pressure, and with contrast-enhanced computed tomography they found a significant reduction in grey matter CBV in response to both stressors (Wilson et al., 2005). Warnert et al. used post exercise ischaemia to induce an increase in SNA, and found a decrease in arterial compliance in the major cerebral arteries at the level of and proximal to the Circle of Willis (Warnert et al., 2015a), and most recently Verbree et al. directly measured reductions in MCA diameter during handgrip exercise using high-resolution MRI (Verbree et al., 2016). These human experiments suggest that SNA has a direct positive effect on vascular resistance in the brain, further supported by evidence showing that blocking SNA doubles the CBF increase associated with a Valsalva manoeuvre (Zhang et al., 2004), but prevents the decrease associated with a head-up tilt (Jordan et al., 2000).

One possible explanation for these data, and the previously observed decreases in CBFV in response to LBNP, is that increased SNA leads to cerebral vasoconstriction, particularly of larger vessels, and LBNP drives an increase in SNA. Perivascular innervation exists primarily with larger arterial vessels and is lost upon entry into the brain parenchyma (Hamel, 2006, Sandor, 1999, Brassard et al., 2017), thus it is eminently plausible that if SNA does lead to vasoconstriction in the human brain, there would be a degree of vessel size dependence in the effect. At a first glance this may seem maladaptive as a response to an orthostatic challenge, but given that increased SNA leads to increased MAP, this could be a protective mechanism to prevent dangerous rises in intracranial pressure. Thus, the systemic autonomic response that serves to raise MAP via peripheral vasoconstriction in order to preserve CPP, may also to some degree effect cerebral arterial vessels too. However, investigating the effect of SNA on cerebral blood vessels was not the main aim of this study, and while the physiological changes we measure are consistent with an increase in SNA, we did not measure this directly and this argument is only speculative.

The other interesting consequence of our interpretation of these data is that they imply that CBV_a_ *increases* in the smallest arterioles, which suggests they are dilating. Due to the vessel size dependent anatomical arrangement of the cer ebral vasculature, this most likely represents dilation in vessels downstream from those that are constricting. This indicates that the increase in overall cerebrovascular resistance is only modest (and thus CBF is relatively preserved) as constriction of larger arterial vessels is offset to some degree by downstream dilation of smaller arterioles. This is in agreement with other studies that found increases in cerebrovascular resistance during LBNP that were relatively small compared with the measured increases in total peripheral resistance (Brown et al., 2003, Levine et al., 1994), and it suggests that this is a result of compensatory autoregulatory mechanisms specific to the brain that lead to downstream vasodilation. A similar phenomenon has also been observed in cats during sympathetic stimulation, whereby CBF is unchanged despite constriction of pial arteries, due to compensatory downstream dilation (Baumbach and Heistad, 1983, Busija et al., 1982).

### Methodological considerations

Previous attempts to estimate CBV_a_ with ASL have often relied upon bipolar gradient pulses to dephase the signal from fast moving spins (“vascular crushing”) (Hoad et al., 2004; Kim and Kim, 2006; Petersen et al., 2006), which can then be used to isolate the intravascular signal if they are applied in an interleaved fashion. As we have not adopted this approach, we are still potentially sensitive to signal from tagged spins that are in exchange with tissue spins, and this effect will be more prevalent in the smaller arterioles whose walls are more permeable. However, by using multiple very short TIs this effect will have minimum contribution to these data, and even the longest TI is earlier than the earliest expected arrival times for tagged blood to the tissue (Macintosh et al., 2010). Additionally, the reduction in CBV_a_ we observed during LBNP points to vasoconstriction of larger arteries, which would suggest a decrease in blood velocity that would be expected to increase tissue arrival time and further reduce the influence of exchange with tissue spins. Thus, it is very unlikely that the increase in CBV_a_ we observe in voxels with low *baseline* CBV_a_ values is a result of an increased signal contribution from a tissue exchange compartment.

The *baseline* dependent response we observe demonstrates the sensitivity of the ASL method to detect changes in arterial blood volume that occur during LBNP. We have proposed a vessel size dependent response as the most plausible explanation, based on *a priori* knowledge of the anatomical arrangement of the cerebral vasculature. However, these result needs to be validated by future studies that used different techniques that allow the response from well defined arterial segments to be separated. Finally, the small sample size and male only subject group demand caution when generalising these findings to the population, and replication in a larger sample would be beneficial.

## Summary

These preliminary data demonstrate the potential for MRI to be used as tool to study the cerebral vascular responses that associated with CA. Typically in the area of research TCD is preferred as it is low cost and easy to implement when gravitational based physiological challenges are employed. However, TCD is only sensitive to blood flow velocity changes in large cerebral arteries for which an acoustic window exists, and so can provide no insight into changes that occur in different segments of the vasculature. The MRI approach we have used here is sensitive to different segments of the arterial vasculature and if combined with an independent measurement of vessel size/type could be even more informative.

This study has combined MRI and LBNP to investigate CA during an orthostatic challenge that induces central hypovolemia. We propose that the data presented are best explained as the result of different segments of the arterial vasculature responding differently to the LBNP challenge, with larger and smaller arterial vessels constricting and dilating, respectively. Finally, this study highlights the potential of MRI as a modality for investigating CA in humans in order to provide a more comprehensive assessment of cerebrovascular health.

## Funding

This work was supported by the Wellcome Trust [WT090199 and WT200804]

## Appendix A

The validity of the brute-force template based fitting procedure was examined as in Wu et al. (2007). 1000 signal time series were randomly selected from a template with parameter values CBV_a_(%) = [0,1, 2, …, 10], BAT(s) = [0.2,0.3,0.4,0.5] and σ(s) = [0,0.2,0.4]s. A reduced range of parameters was chosen for BAT to ensure the simulated BAT was always in the range of experimental TIs (0.15–0.6), i.e. only simulating the first slice acquired to avoid the complexity of dealing with slice timings. Randomly selected template time courses were superimposed with Gaussian noise with SNR = 10, as is typical for data acquired with this PICORE sequence (Wu et al., 2007). The mean absolute variation from the true value in parameter estimates was ~ 0.67% for CBVa, ~ 0.11 s for BAT, and ~ 0.066 s for dispersion σ. Thus, the overall error in CBV_a_ estimates was less than the 1% discrete values used for the fit, which means we can be confident that the fitting approach is robust, and the observed effect is not due to inaccurate CBV_a_ estimation. As expected, the course temporal resolution resulted in a larger error in BAT estimation.
