# Supplementary material for: Changes in arterial cerebral blood volume during lower body negative pressure measured with MRI
Source: Neuroimage. 2019 Feb 15;187:166–75. doi: 10.1016/j.neuroimage.2017.06.041 (PMC6414398; doi:10.1016/j.neuroimage.2017.06.041)
Supplement: Supplementary file 1 — Supplementary material [file mmc1.pdf]

Supplementary material

A

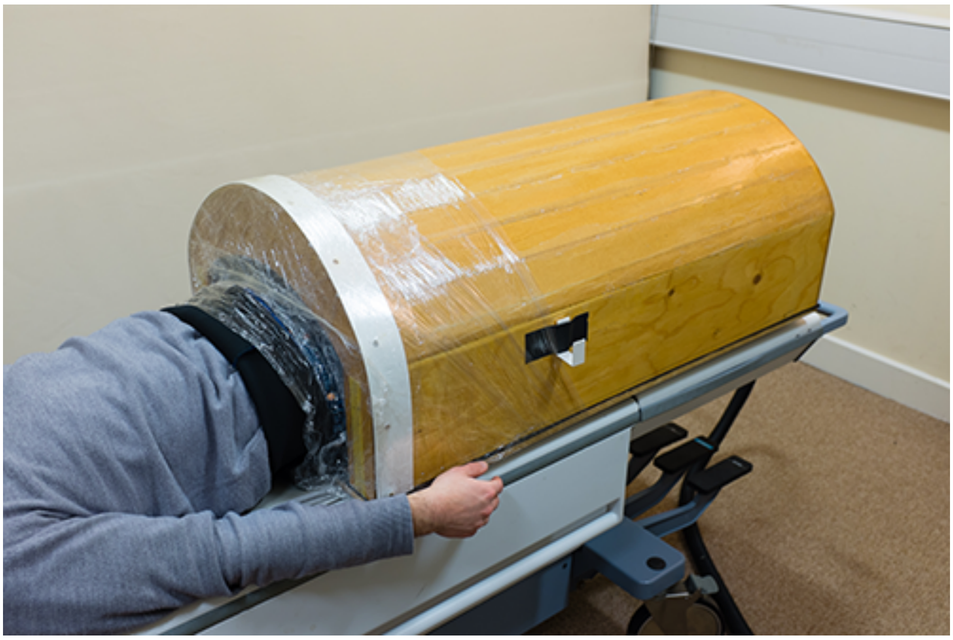

B

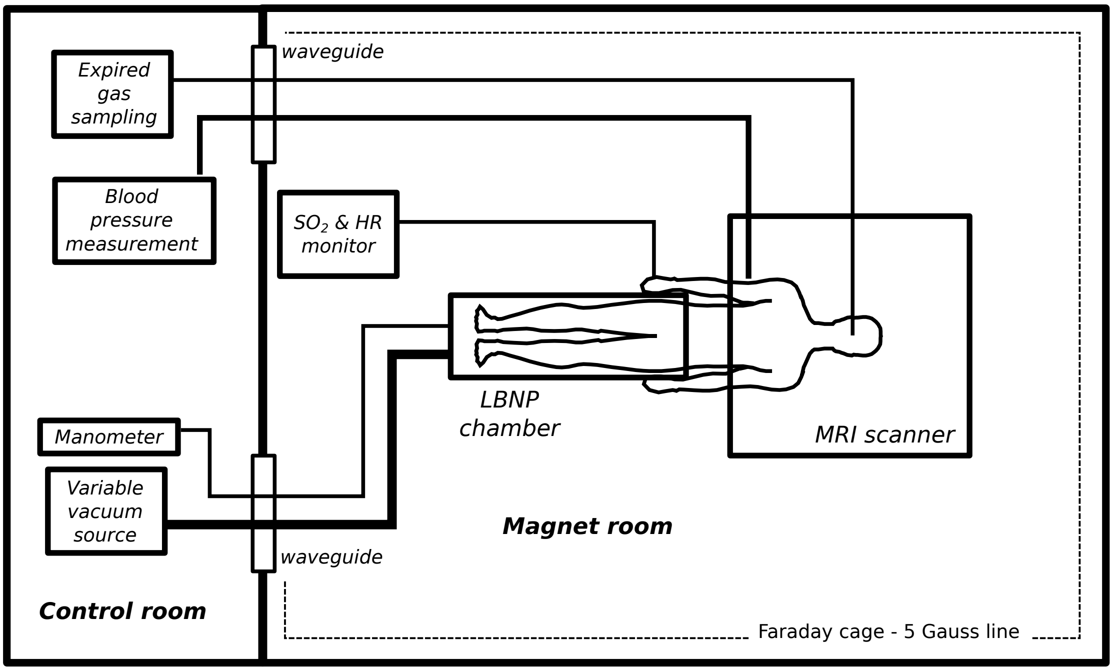

Figure 1. A) A photograph of the MRI compatible LBNP chamber. Plastic wrap was used to make create an airtight seal sufficient to maintain a negative pressure of -40 mmHg. B) A schematic showing the experimental set-up of the LBNP chamber in the MR environment.

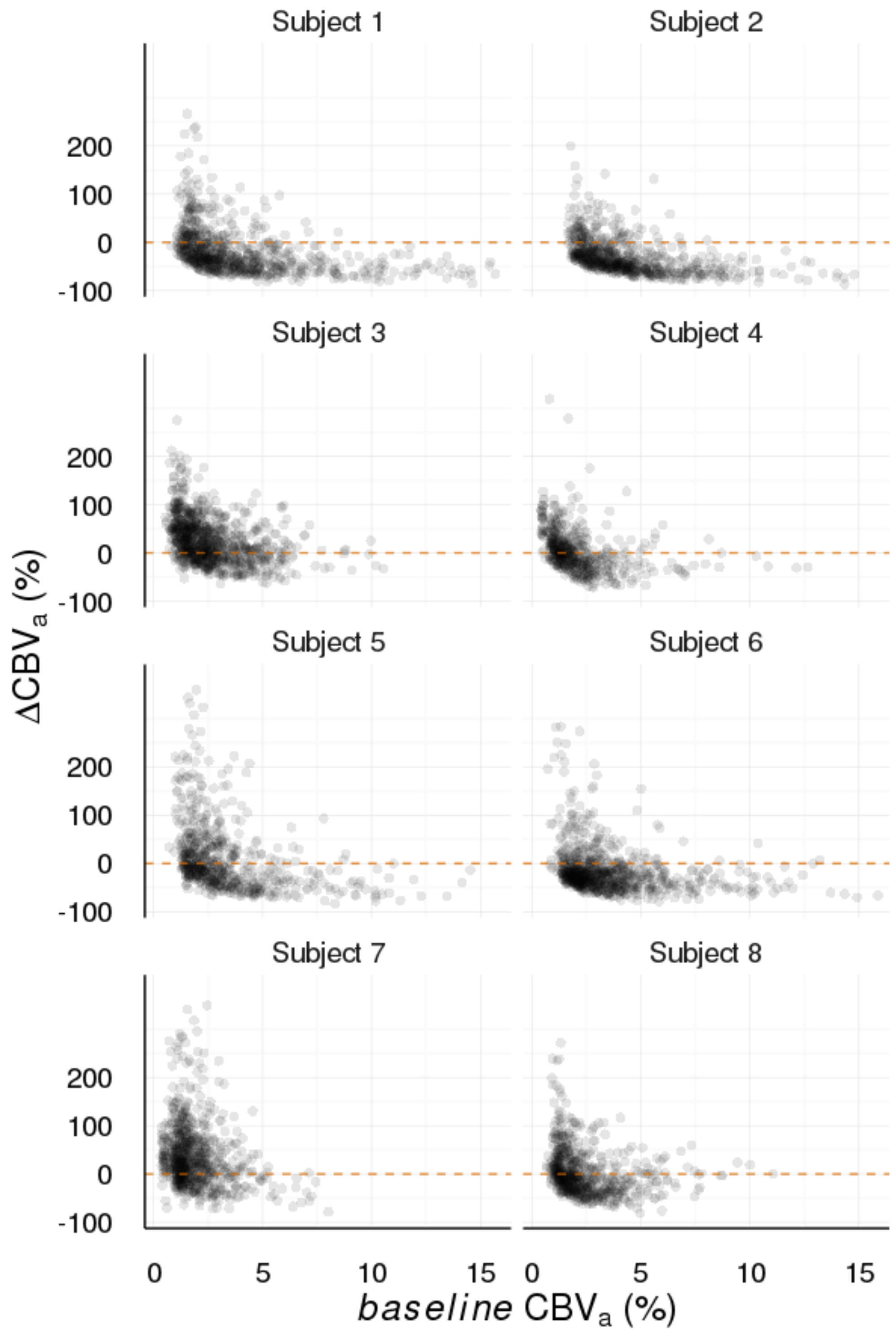

Figure 2. For each subject, fractional changes in CBV_a during *lbnp* (%ΔCBV_a) plotted as a function of *baseline* CBV_a.

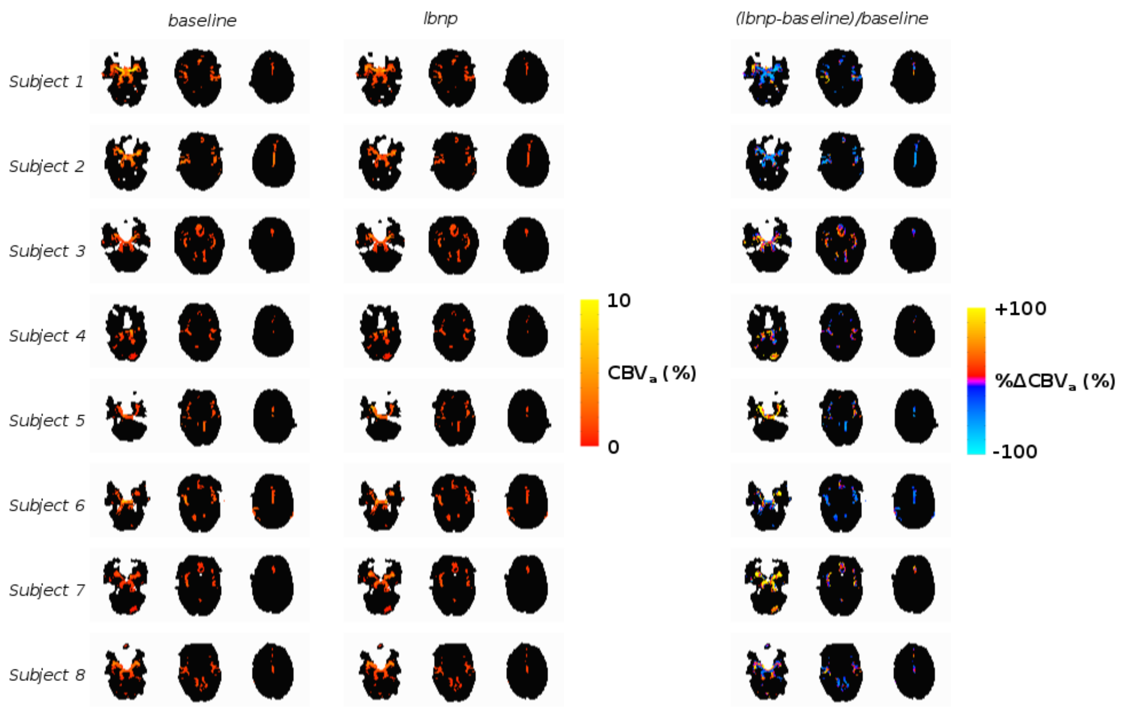

Figure 3. Individual subjects' TI4 estimate CBV$_a$ maps for both conditions and the fraction change between conditions.
